# Curcumin Ameliorates DSS-Induced Colitis in Mice Through Modulation of Gut Microbiota and Metabolites

**DOI:** 10.3390/life15071153

**Published:** 2025-07-21

**Authors:** Chengxue Yi, Yuxuan Xia, Jiajing Yan, Wen Xia, Haoyu Wang, Fei Mao, Pan Huang

**Affiliations:** 1 School of Medical Technology, Zhenjiang College, Zhenjiang 212028, China; 2 School of Medicine, Jiangsu University, Zhenjiang 212013, China

**Keywords:** curcumin, colitis, gut microbiota, metabolites, mouse

## Abstract

In this study, we established a mouse colitis model using DSS to investigate the impact of curcumin on gut injury, the intestinal microbiota, and fecal metabolites. The findings indicated that curcumin effectively mitigated weight loss and colon shortening caused by colitis, enhanced the expression of anti-inflammatory factor *IL-10* mRNA (*p* < 0.05), and suppressed the expression of pro-inflammatory factors (*IL-1β*, *IL-6*, and *TNF-α* mRNA; *p* < 0.05). 16S rDNA sequencing analysis showed that in the CUR group, compared to the NC and DSS groups, the abundances of Bacteroides, Lachnospiraceae NK4A136, and Ruminococcaceae UGC 014 significantly increased, while that of Lactobacillus markedly decreased. Additionally, compared with the DSS group, the CUR group demonstrated a significant decrease in levels of metabolites associated with nucleic acid and fat metabolism, including xanthosine, isocitric acid, and D-xylose. Conversely, levels of metabolites of curcumin, such as demethoxycurcumin and tetrahydrocurcumin, were significantly elevated in the CUR group. Curcumin appears to offer protection against mouse colitis by potentially enhancing the composition of the gut microbiota and regulating metabolic and inflammatory processes through its metabolites.

## 1. Introduction

Ulcerative colitis (UC) is a chronic, non-specific inflammatory disorder characterized by lesions primarily in the mucosa and submucosa of the rectum and colon [1]. These continuously distributed lesions initially present as punctate hemorrhages and small abscesses in the mucosa, which gradually enlarge and progress to mucosal necrosis, sloughing, and ulcer formation, characteristic of one of the types of inflammatory bowel disease (IBD) [2]. Current primary treatments for UC, including 5-aminosalicylates, corticosteroids, immunosuppressants, and antibiotics, commonly induce adverse effects such as acne, abdominal pain, weight gain, diabetes, and elevated blood pressure [3,4]. In recent years, both the incidence and prevalence of UC in China have risen sharply, with an annual incidence rate of approximately 1.18 per 100,000 and a prevalence rate of about 11.6 per 100,000 [5]. UC represents a significant health threat, and exploring its pathogenesis and identifying new targets for prevention and treatment are crucial. Currently, the development of UC is attributed to a combination of factors, including genetics, immunity, the gut microbiota, and the environment. Of these, dysbiosis of the gut microbiota is viewed as a principal environmental contributor to UC [6,7]. Plentiful research has shown that the composition and functional characteristics of the gut microbiota have crucial roles in maintaining the physiological functions of the colon [8]. Certain probiotics are capable of protecting the colon from inflammatory processes, while an increase in the abundance of pathogens in the colon is correlated with the risk for IBD [9,10].

Curcumin (CUR), a hydrophobic polyphenol, is derived from the rhizomes of ginger plants and appears as a yellowish powder. It is extensively used as a pigment and food additive. Owing to its properties, including antioxidant, anti-inflammatory, anti-tumor effects, and capability for mucosal repair—combined with its low toxicity and good tolerance—curcumin has garnered significant interest for its potential in treating UC in recent years [11,12]. Curcumin significantly reduces the levels of pro-inflammatory cytokines involved in the onset and progression of UC, such as IL-1α, IL-1β, IL-6, IL-8, IL-23, and TNF-α, among others [13], and it also modulates inflammation by directly inhibiting key signaling pathways, including mitogen-activated protein kinase (MAPK), nuclear factor kappa-B (NF-κB), and toll-like receptor 4 (TLR-4) [14]. Additionally, curcumin interacts with the intestinal microbiota to enhance gut health. It is metabolized by the gut microbiota into small molecules that facilitate absorption [15,16], while also promoting the growth of beneficial bacterial strains, boosting microbial diversity, and strengthening the intestinal barrier [15,17].

Extensive research has predominantly concentrated on elucidating the molecular mechanisms underlying curcumin’s immunomodulatory properties, with a consensus emerging regarding its potent anti-colitis efficacy [18,19]. While several investigations have examined the relationship between curcumin-induced gut microbiota alterations and IBD pathogenesis [20], the influence of curcumin intervention on gut microbial metabolites remains markedly understudied in the current literature. This study seeks to establish a DSS-induced mouse colitis model and explore the effects of curcumin on intestinal inflammation, the gut microbiota, and fecal metabolites in mice with colitis.

## 2. Results

### 2.1. The Effects of Curcumin on Mice Body Weight and DAI in Mice

**Figure 1:**
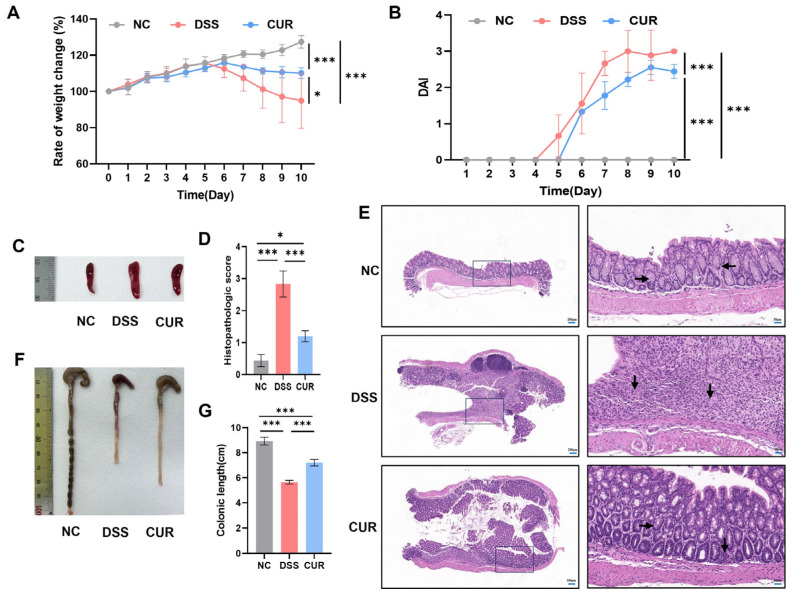
The effect of curcumin on mice body weight, DAI, and histopathology in mice. (**A**) Changes in body weight of mice; (**B**) disease activity index (DAI); (**C**) spleen size; (**D**) histopathological score; (**E**) histopathology of colon; (**F**) colon length measurements; (**G**) colon length analysis. →/←: the horizontal arrow indicates the digestive gland; ↓: the downward-pointing arrow indicates the inflammatory exudation. NC: negative control group; DSS: DSS−treated group; CUR: DSS + CUR−treated group. Statistical significance markers are defined as * *p* < 0.05, ** *p* < 0.01, and *** *p* < 0.001.

As illustrated in Figure 1A, there was a notable reduction in body weight observed in both the DSS and CUR groups by day 6 compared to the NC group (*p* < 0.05); however, this decrease was slower in the CUR group. Figure 1B shows the temporal progression of disease activity index (DAI) scores, calculated through stool consistency, rectal bleeding, and body weight changes. The DSS group exhibited significant disease-associated symptoms starting from day 4, with DAI scores reaching peak severity (3.0 ± 0.577) on day 8 and sustained elevated levels thereafter. By contrast, the CUR group manifested delayed symptom onset from day 5, achieving a lower peak DAI value (2.6 ± 0.191) on day 9 followed by a decline (2.4 ± 0.191). The CUR group exhibited a delay in disease progression when compared to the DSS group, and the peak value of the DAI was effectively reduced during the acute phase. Evidenced by the mitigation of weight loss and the alleviation of hematochezia symptoms, the CUR group demonstrated a notable capacity to promote disease remission.

### 2.2. The Effects of Curcumin on Spleen Size, Colon Length, and Histopathology in Mice

Figure 1C reveals that, compared to the NC group, significant splenomegaly was observed in both the DSS and CUR groups, with the spleen size being smaller in the CUR group than in the DSS group. Colon length measurements demonstrated distinct variations across experimental groups (Figure 1F,G). The NC group exhibited the longest colons (8.93 ± 0.29 cm), consistent with intact intestinal morphology. By contrast, DSS-treated mice displayed significant colon shortening (5.63 ± 0.17 cm), a hallmark of colitis-associated tissue damage. CUR intervention partially ameliorated this reduction, with colon lengths (7.20 ± 0.26 cm) significantly longer than in the DSS group yet shorter than in the NC group. Histopathological examination and quantitative scoring of colonic tissues revealed significant differences among experimental groups (Figure 1D,E). The NC group exhibited intact mucosal architecture with regularly arranged crypts, tightly connected epithelial cells, and the absence of inflammatory cell infiltration or edema. By contrast, DSS-treated mice displayed typical colitis pathology, characterized by extensive mucosal erosion, disrupted crypt architecture, disorganized epithelial cell alignment, and dense inflammatory cell infiltration. CUR intervention partially restored mucosal integrity, improved crypt regularity compared to the DSS group, and reduced inflammatory cell infiltration, suggesting its reparative effects on colitis-induced damage. Quantitative histopathological scoring further corroborated these observations: the DSS group showed significantly elevated scores (2.83 ± 0.404) versus the NC group (0.44 ± 0.191), while CUR treatment markedly attenuated tissue damage (1.2 ± 0.173). These findings collectively indicated that DSS successfully induced colitis-associated histopathological changes, whereas CUR mitigated mucosal injury through partial restoration of barrier integrity and anti-inflammatory mechanisms.

### 2.3. The Effects of Curcumin on Inflammatory Factors in the Colon of Mice

**Figure 2:**
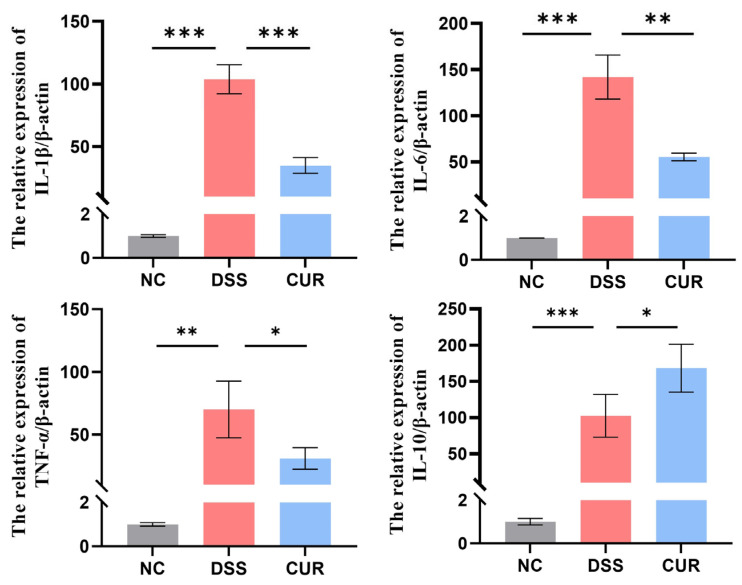
The effects of curcumin on inflammatory factors in the colon of mice. NC: negative control group; DSS: DSS−treated group; CUR: DSS + CUR−treated group. Statistical significance markers are defined as * *p* < 0.05, ** *p* < 0.01, and *** *p* < 0.001.

As depicted in Figure 2, RNA was extracted from colonic tissues, and q-PCR analysis was performed to evaluate mRNA expression (*IL-1β*, *IL-6*, *TNF-α*, and *IL-10*). The results showed that, compared to the NC group, there were significantly elevated levels of *IL-1β*, *IL-6*, *TNF-α*, and *IL-10* mRNA in both the DSS and CUR groups (*p* < 0.05). Notably, the expression levels of *IL-1β*, *IL-6*, and *TNF-α* mRNA were significantly higher in the DSS group than in the CUR group (*p* < 0.05), while *IL-10* mRNA expression was significantly higher in the CUR group than in the DSS group (*p* < 0.05).

### 2.4. The Effect of Curcumin on Gut Microbiota Diversity

**Figure 3:**
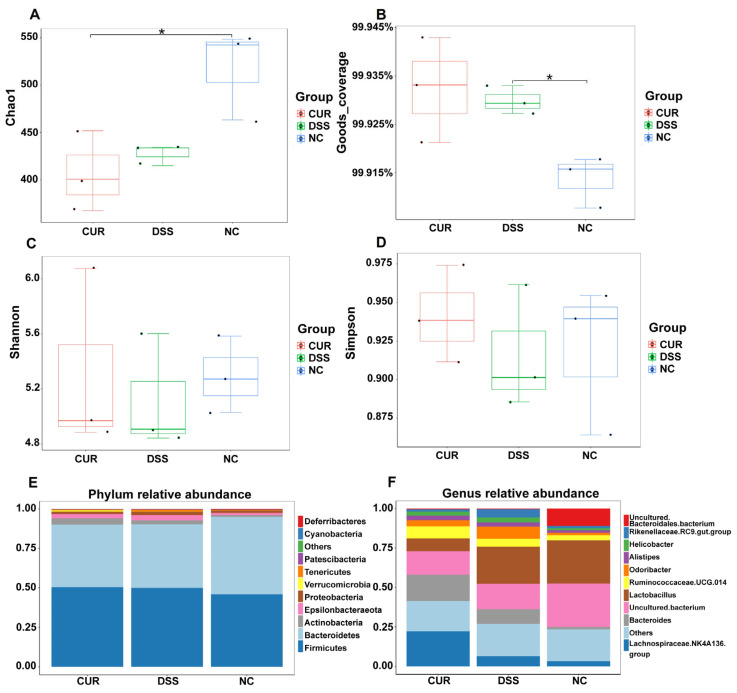
The effect of curcumin on the gut microbiota. (**A**) Chao1; (**B**) Goods coverage; (**C**) Shannon index; (**D**) Simpson index; (**E**) phylum; (**F**) genus. NC: negative control group; DSS: DSS−treated group; CUR: DSS + CUR−treated group. Statistical significance markers are defined as * *p* < 0.05, ** *p* < 0.01, and *** *p* < 0.001.

Several alpha diversity indices were measured to assess changes in gut microbiota diversity among the NC, DSS, and CUR groups. Figure 3A illustrates that the diversity of the gut microbiota, estimated using the Chao1 index, was lower in both the DSS and CUR groups compared to the NC group, with the DSS group exhibiting higher diversity than the CUR group. Figure 3B indicates that Goods coverage was above 0.9915 in all three groups, suggesting high sampling quality and that the sequencing results accurately reflected the actual conditions of the samples. Figure 3C,D, utilizing the Shannon and Simpson indices, show that compared to the NC group, the microbiota compositions in both the DSS and CUR groups were less diverse, with a significant reduction in microbial diversity. However, the Shannon index was higher in the CUR group than in the DSS group, indicating that curcumin may play a role in preventing the reduction of microbial diversity induced by DSS.

### 2.5. The Effect of Curcumin on Gut Microbiota Species Abundance

Figure 3E demonstrates that at the phylum level, compared to the NC group, the abundance of *Bacteroidetes (Bacteroides)* in both the CUR and DSS groups significantly decreased, while the abundances of *Verrucomicrobia* and *Actinobacteria* significantly increased in the CUR group relative to both the NC and DSS groups. Figure 3F, analyzing at the genus level, shows that compared to the NC group, the abundances of *uncultured bacterium* and *uncultured bacteroidales bacterium* in the DSS and CUR groups notably decreased. Notably, compared to the NC and DSS groups, the abundances of *Bacteroides, Lachnospiraceae NK4A136*, and *Ruminococcaceae UGC 014* in the CUR group notably increased, while the abundance of *Lactobacillus* significantly decreased.

### 2.6. Analysis of Major Components of Fecal Metabolites

**Figure 4:**
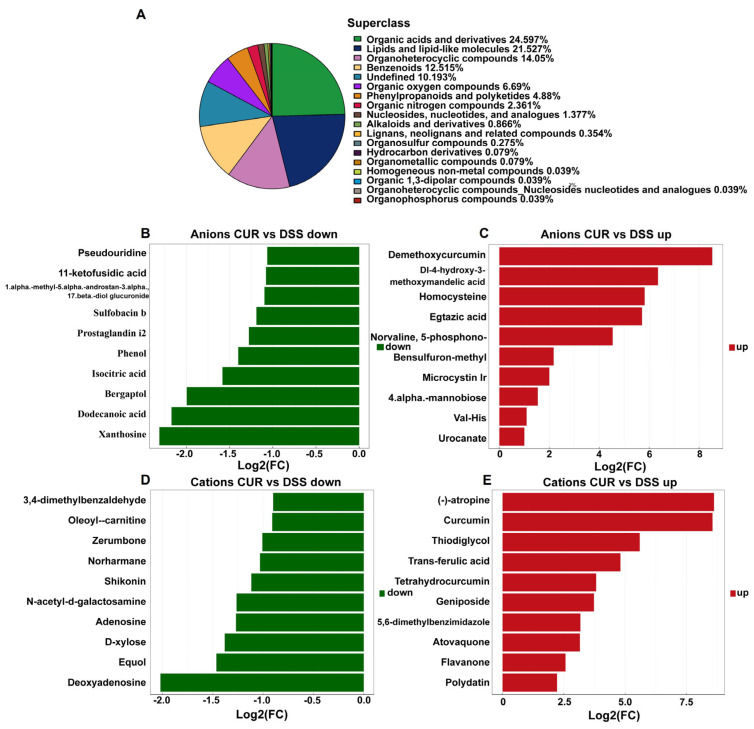
Differential metabolite analysis of feces. (**A**) Major components of fecal metabolites; (**B**) anions CUR vs. DSS down; (**C**) anions CUR vs. DSS up; (**D**) cations CUR vs. DSS down; (**E**) cations CUR vs. DSS up. NC: negative control group; DSS: DSS−treated group; CUR: DSS + CUR−treated group.

As illustrated in Figure 4A, most substances in mouse feces were organic compounds and their derivatives, including phospholipids and related compounds. Specifically, organic acids and derivatives comprised 24.597%, lipids and lipid-like molecules comprised 21.527%, organ heterocyclic compounds comprised 14.05%, benzenoids comprised 12.515%, undefined substances comprised 10.193%, and organic oxygen compounds comprised 6.69%.

### 2.7. Differential Metabolite Analysis of Feces

As depicted in Figure 4B–E, differential analysis of metabolites in mouse feces between the DSS and CUR groups showed that compared to the DSS group, the CUR group exhibited significant decreases in levels of anions such as xanthosine, dodecanoic acid, bergaptol, isocitric acid, and phenol, along with cations including deoxyadenosine, equol, D-xylose, adenosine, and N-acetyl-D-galactosamine. Conversely, increased levels were observed for anions like demethoxycurcumin, Dl-4-hydroxy-3-methoxymandelic acid, homocysteine, egtazic acid, norvaline, and 5-phosphono-, as well as cations such as (−)-atropine, curcumin, thiodiglycol, trans-ferulic acid, and tetrahydrocurcumin.

## 3. Discussion

Curcumin, a major component of curcuminoids, is extensively utilized in the food industry and has been recognized for its significant anti-inflammatory characteristics, particularly in treating chronic inflammatory underlying diseases [21]. Consequently, our study employed a mouse model of DSS-induced colitis to investigate the effects of curcumin on IBD and its underlying mechanisms. Our findings indicated that curcumin mitigates colitis-induced symptoms, including weight loss, increased DAI scores, spleen enlargement, shortened colon length, and colon structure damage. Consistent with our results, Liu et al. reported that turmeric-derived nanoparticles could alleviate colon structure damage and colitis by inhibiting the NF-κB and STAT_3_ signaling pathways [22,23].

Additionally, our study demonstrates that curcumin upregulates the expression of *IL-10* mRNA and downregulates the expression of *IL-1β*, *IL-6*, and *TNF-α* mRNA in the colon of colitic mice. Curcumin is known to inhibit I kappa B (IκB) kinase, thereby preventing cytokine-mediated phosphorylation and degradation of IκB, which inhibits NF-κB and reduces the expression of pro-inflammatory cytokines [24,25,26]. On the contrary, studies have found that curcumin supplementation induced mild anemia, reduced iron stores, exacerbated colitis, and markedly reduced overall survival [27].

In our study, 16S rDNA gene sequencing was used to analyze the intestinal microbiota in fecal samples, and raw sequencing reads were demultiplexed and trimmed of adapters/barcodes. Denoising and generation of amplicon sequence variants (ASVs) were performed using the DADA2 pipeline within QIIME2. Taxonomic annotation of ASVs was conducted via the classify-sklearn module against the SILVA 138 reference database. Microbial community composition at both phylum and genus levels was calculated based on relative abundance. Stacked bar plots were generated to visualize the top 10 most abundant taxa at each taxonomic rank. The results revealed that curcumin modifies the composition of the intestinal microbiota, enhancing the abundances of Bacteroides, *Staphylococcus*, and *Lachnospiraceae NK4A136*. In contrast to our findings, Guo et al. reported that curcumin effectively modulates the abundances of specific bacterial genera, including *Akkermansia, Coprococcus, Roseburia*, and *Turicibacter*, as well as bacterial families such as *F16, Enterococcaceae,* and *Aerococcaceae*. However, no significant changes were observed in the abundances of *Bacteroides, Staphylococcus,* and several other taxa [20]. Prior research has demonstrated that *Bacteroides* can mitigate DSS-induced colitis, and *Bacteroides*-derived sphingolipids, known to be significantly reduced in the feces of IBD patients, are inversely associated with intestinal inflammation [28,29,30]. These sphingolipids also play a role in the colonization of symbiotic bacteria and help to reduce microbiota dysbiosis [28]. Furthermore, studies have shown that *Bacteroides* can suppress NF-κB signaling in the colon, thereby decreasing serum TNF-α levels [31]. Therefore, curcumin may alleviate IBD by inhibiting IκB kinase and reducing the abundance of *Bacteroides*, which in turn may suppress NF-κB activation and mitigate inflammation in IBD. Additionally, metabolites derived from *Bacteroides*, such as 3-ASA, CDCA, and 4-HPAA, are reported to possess anti-inflammatory properties [32]. *Lachnospiraceae NK4A136*, part of the family *Lachnospiraceae*, is an anaerobic, spore-forming bacterium that produces short-chain fatty acids (SCFAs) through the fermentation of dietary polysaccharides and is considered a potential probiotic. It is inversely associated with several metabolic diseases and chronic inflammation [33,34,35,36].

Several studies have highlighted the intricate crosstalk between curcumin and gut microbial composition [37]. One of the key mechanisms by which curcumin affects the gut microbiome lies in its capacity to regulate microbial diversity and abundance. Xiao et al. demonstrated that curcumin can restore homeostasis in Th17/Treg responses within the gut, thereby modulating the gut microbiota composition in mice with diabetic complications [38]. Conversely, growing evidence suggests that the gut microbiome itself plays a pivotal role in regulating the bioavailability, metabolism, and therapeutic effects of curcumin within the body [37].

Intestinal feces play a crucial role in IBD by participating in various host metabolic pathways and producing fermentation metabolites. In this study, intestinal metabolomics in mice were analyzed using LC-MS. Compared to the DSS group, levels of homocysteine, (−)-atropine, and trans-ferulic acid were elevated in the CUR group, whereas levels of xanthosine, isocitric acid, and D-xylose decreased. Notably, trans-ferulic acid, a principal polyphenolic compound found in oleoresin brines, possesses antioxidant and anti-inflammatory properties [39]. Aligning with our findings, other studies have indicated that trans-ferulic acid could be developed as a potential therapy for multiple diseases, including cardiovascular disorders, Alzheimer’s disease, and diabetes mellitus, and it has a protective effect on the intestinal epithelial barrier by enhancing the expression of tight junction (TJ) proteins, including claudin-1, occludin, and ZO-1, in intestinal epithelial cells [40,41,42,43,44]. Moreover, compared to the DSS group, levels of metabolites of curcumin such as demethoxycurcumin and tetrahydrocurcumin showed significant increases in the CUR group. These compounds, active components of turmeric, are linked to intestinal inflammation and are known for their anti-inflammatory and antioxidant properties, potentially alleviating symptoms associated with intestinal inflammation [45,46,47].

## 4. Materials and Methods

### 4.1. Animal Experiment Protocol

The study was approved by the Ethical Committee of Jiangsu University (2024051102). The Center of Animal Laboratory at Jiangsu University (Zhenjiang, China) provided male C57BL/6J mice, each weighing approximately 23 g and aged 9 weeks. These mice were acclimatized in a pathogen-free laboratory environment maintained at 22 ± 3 °C with 40–60% humidity. We randomly assigned the mice into three groups, each comprising 10 mice, and observed them over a 10 day period. Mice exhibiting a rapid loss of 15–20% body weight, a complete loss of appetite for 24 h or poor appetite (less than 50% of normal) for three consecutive days, and the inability to feed and drink independently were euthanized. While the control group (NC) received demineralized water, the DSS and CUR groups were administered 2% DSS (CAS:9011-18-1, Meilun, Dalian, China) in their drinking water for seven days. The drinking water for the CUR group also contained 0.5 mg/mL of curcumin (CAS:458-37-7, ≥95%, Macklin, Shanghai, China). The drinking water was replaced daily, and water consumption was recorded. On the eighth day, the DSS group’s water was replaced with demineralized water, while the CUR group continued with demineralized water containing 0.5 mg/mL of curcumin. On the 10th day, all mice were euthanized.

The mice were fed standard food and later euthanized within 10 days. They were anesthetized using pentobarbital (40 mg/kg), and approximately 500 µL of blood was collected via orbital puncture. Serum was separated by centrifugation at 3000 rpm for 15 min at 4 °C. Subsequently, the mice were euthanized by carbon dioxide (30–70%) asphyxiation. If the mice exhibited no movement, no breathing, and dilated pupils, the carbon dioxide valve was turned off and the mice were observed for an additional 2–3 min to confirm their deaths. Prior to storage, colon tissues were divided and stored in a freezer at −80 °C. Intestinal feces were removed before freezing at −80 °C for 16S rDNA sequencing and stored in a liquid nitrogen tank.

### 4.2. Analysis of Morphology

Phosphate-buffered solution (PBS) was used to wash paraformaldehyde (4%)-fixed tissues before sequential dehydration with ethanol (70, 95, and 100%) and subsequent treatment with anhydrous alcohol. Following clarification with xylene, the colon tissues were sectioned (5 µm thick) and embedded in paraffin. Staining of these sections was performed using hematoxylin and eosin (H&E) (SolarBio, Beijing, China) [48,49].

### 4.3. RNA Extraction, cDNA Synthesis, and Real-Time Polymerase Chain Reaction (PCR) Analysis

For tissue analysis, colon specimens were first frozen in liquid nitrogen, then pulverized and mixed with TRIzol reagent (Ambion, Waltham, MA, USA). Total RNA extraction from the tissues was carried out following the manufacturer’s protocol for TRIzol reagent. The concentration and quality of RNA were measured using a NanoDrop Spectrophotometer (Thermo Fisher Scientific, Waltham, MA, USA), followed by reverse transcription of RNA into cDNA using the High-Capacity cDNA Reverse Transcription Kit (Applied Biosystems, Foster City, CA, USA), as per the manufacturer’s instructions. Quantitative real-time PCR (qRT-PCR) was performed on the DNA Engine Opticon system (Bio-Rad Laboratories, Hercules, CA, USA) using Maxima SYBR-green Master Mix (Thermo Fisher Scientific). The primer sequences are detailed in Table 1. For the quantification of target gene expression, β-actin served as the reference gene for normalization, and relative expression levels compared to control mice were calculated using the 2^−ΔΔCt^ method [50,51].

### 4.4. Analysis of Gut Microbiota

Following the manufacturer’s protocol, we sequenced samples using the Illumina NovaSeq platform provided by LC-Bio (Hangzhou, China). To assess species diversity, we calculated alpha diversity using five indicators: observed species, Good’s coverage, Chao1, Simpson, and Shannon, utilizing QIIME2 for these calculations. Beta diversity was also analyzed with QIIME2, and data visualization was performed using R. Sequence alignment was conducted with BLAST v2.15.0, using the SILVA database to annotate each representative sequence. Additional visualizations were generated using R version 3.5.2 [9].

### 4.5. Fecal Sample Preparation for Metabolomics Analysis

Metabolites were extracted by adding 1 mL cold methanol/acetonitrile/H_2_O (2:2:1, *v*/*v*/*v*) to 80 mg sample, vortexing, incubating on ice for 20 min, and centrifuged at 14,000 g for 20 min at 4 °C. The supernatant was filtered through a 96-well protein precipitation plate, eluted, dried under vacuum at 4 °C, reconstituted in 100 μL acetonitrile/water (1:1, *v*/*v*), and transferred to LC vials for LC-MS analysis [52].

### 4.6. LC-MS Analysis

Untargeted metabolomics analysis of polar metabolites was performed via UPLC-ESI-QTOF-MS (Sciex TripleTOF 6600) at Shanghai Applied Protein Technology. Samples were separated on an ACQUITY UPLC BEH amide column (2.1 mm × 100 mm, 1.7 μm) using a gradient of solvent A (25 mM ammonium acetate/25 mM ammonium hydroxide in water) and solvent B (acetonitrile). The gradient program was: 85% B (1 min) → 65% B (11 min) → 40% B (4 min) → 85% B (5 min re-equilibration). The LC conditions were: flow rate 0.4 mL/min, column temperature 25 °C, autosampler temperature 5 °C, and injection volume 2 μL. MS was operated in both ionization modes with the parameters detailed in Table 2.

### 4.7. Statistical Analysis

Data in this study are expressed as the mean ± SD, and statistical analysis was performed using GraphPad Prism 8.0. Statistical analyses were conducted as follows: body weight and disease activity were evaluated using two-way analysis of variance (ANOVA), while the remaining data were analyzed via one-way ANOVA, with post hoc comparisons performed using the Tukey multiple comparison test. Statistical significance markers are defined as * *p* < 0.05, ** *p* < 0.01, and *** *p* < 0.001, and all chart error lines represent SD values.

## 5. Conclusions

Curcumin, a potent antioxidant and anti-inflammatory compound, has attracted considerable attention for its therapeutic potential. Our findings suggest that curcumin may exert protective effects against DSS-induced colitis in mice by modulating the gut microbiota and its metabolites. These results offer new insights into the functional properties of curcumin and its potential clinical application in UC treatment. However, further studies incorporating fecal microbiota transplantation (FMT) or antibiotic-treated models are needed to better distinguish causation from correlation. Additionally, this study has several limitations. The relatively small sample size may limit the generalizability of the findings. Furthermore, the molecular mechanisms by which curcumin modulates the gut microbiota and inflammatory responses remain to be fully elucidated. Another concern is the known instability of curcumin in aqueous solution under light exposure, which may affect its bioavailability. Although we minimized this issue by replacing the curcumin-containing water daily, the possibility of degradation cannot be entirely excluded. Further studies are warranted to clarify these mechanisms and to optimize both the formulation and stability of curcumin for future applications.

## Figures and Tables

**Table 1:** Sequences of primers used in qRT-PCR.

Gene	Forward	Reverse
Mouse *β-actin*	CCCGCGAGTACAACCTTCTTG	ACCCATACCCACCATCACAC
Mouse *IL-1β*	ATGCCACCTTTTGACAGTGATG	TGATGTGCTGCTGCGAGATT
Mouse *IL-6*	TTTCCTCTGGTCTTCTGGAGT	TCTGTGACTCCAGCTTATCTCTTG
Mouse *IL-10*	TGAATTCCCTGGGTGAGAAGC	CACCTTGGTCTTGGAGCTTATT
Mouse *TNF-α*	CCCTCACACTCACAAACCAC	ACAAGGTACAACCCATCGGC

**Table 2:** MS parameters for untargeted metabolomics analysis.

Parameter Category	Positive Mode	Negative Mode
ESI Source	Gas1/Gas2/CUR = 60/60/30 Temp = 600 °C ISVF = +5500 V	Gas1/Gas2/CUR = 60/60/30 Temp = 600 °C ISVF = −5500 V
MS Acquisition	*m/z* 60–1000 Da Accumulation time = 0.20 s/spectrum	*m/z* 60–1000 Da Accumulation time = 0.20 s/spectrum
MS/MS (IDA Mode)	*m/z* 25–1000 Da Accumulation time = 0.05 s/spectrum CE = 35 V ± 15 eV DP = +60 V Isotope exclusion = 4 Da Ions/cycle = 10	*m/z* 25–1000 Da Accumulation time = 0.05 s/spectrum CE = 35 V ± 15 eV DP = −60 V Isotope exclusion = 4 Da Ions/cycle = 10

## Data Availability

The data generated in the present study may be requested from the corresponding author.
